# Identification of birch lncRNAs and mRNAs responding to salt stress and characterization of functions of lncRNA

**DOI:** 10.1093/hr/uhac277

**Published:** 2022-12-09

**Authors:** Yaqi Jia, Huimin Zhao, Yani Niu, Yucheng Wang

**Affiliations:** State Key Laboratory of Tree Genetics and Breeding, Northeast Forestry University, 26 Hexing Road, Harbin 150040, China; State Key Laboratory of Tree Genetics and Breeding, Northeast Forestry University, 26 Hexing Road, Harbin 150040, China; State Key Laboratory of Tree Genetics and Breeding, Northeast Forestry University, 26 Hexing Road, Harbin 150040, China; State Key Laboratory of Tree Genetics and Breeding, Northeast Forestry University, 26 Hexing Road, Harbin 150040, China; Key Laboratory of Forest Tree Genetic Breeding and Cultivation of Liaoning Province, Shenyang Agricultural University, 120 Dongling Road, Shenyang 110866, China

## Abstract

Long noncoding RNAs (lncRNAs) are important in abiotic stress tolerance. Here, we identified salt-responsive genes and lncRNAs in the roots and leaves of *Betula platyphylla* Suk. (birch), and characterized their lncRNAs functions. In total, 2660 mRNAs and 539 lncRNAs responding to salt treatment were identified using RNA-seq. The salt-responsive genes were substantially enriched in ‘cell wall biogenesis’ and ‘wood development’ in the roots and were enriched in ‘photosynthesis’ and ‘response to stimulus’ in the leaves. Meanwhile, the potential target genes of the salt-responsive lncRNAs in roots and leaves were both enriched in ‘nitrogen compound metabolic process’ and ‘response to stimulus’. We further built a method for quickly identifying abiotic stress tolerance of lncRNAs, which employed transient transformation for overexpression and knock-down of the lncRNA, enabling gain- and loss-of-function analysis. Using this method, 11 randomly selected salt-responsive lncRNAs were characterized. Among them, six lncRNAs confer salt tolerance, two lncRNAs confer salt sensitivity, and the other three lncRNAs are not involved in salt tolerance. In addition, a lncRNA, *LncY1*, was further characterized, which improves salt tolerance by regulating two transcription factors, *BpMYB96* and *BpCDF3*. Taken together, our results suggested that lncRNAs play important roles in the salt response of birch plants.

## Introduction

Identifying genes or long noncoding RNAs (lncRNAs) responding to abiotic stress on genome-scale is important in revealing the stress-tolerant mechanism. RNA sequencing (RNA-seq) technology enables analysis of gene expression on genome-scale, which also could efficiently identify ncRNAs from various organisms [1]. LncRNAs refer to RNAs that are more than 200 nt in length, lack open reading frames (ORFs), and do not encode proteins. Similar to mRNAs, most lncRNAs in plant or animal genomes are transcribed by RNA polymerase II, although lncRNAs also can be transcribed by RNA polymerase III, IV, or V [2, 3]. Some lncRNAs also comprise a 5′ cap and 3′ polyadenylated tail, yet still do not encode proteins [3]. In the genome, lncRNAs are typically transcribed from intronic and intergenic regions, and the antisense or the sense strands of coding genes. Therefore, according to their position in the genome, they are classified into three groups, comprising incRNAs (intronic ncRNAs), NATs (natural antisense transcripts), and lincRNAs (long intergenic non-coding RNAs) [4, 5]. LncRNA transcriptomes are unique to each species. For instance, the similarity in mRNAs between mice and humans is 92%; but the similarity of lncRNAs between them is only 35% [6]. Similar phenomena were observed in plant species. For instance, no more than 0.4% of lncRNAs were found to be commonly expressed in two different tomato species [7]. These findings suggested the complex origins of these species-specific lncRNAs in genomes. LncRNAs were found to play diverse regulatory roles in transcription, post-transcription, translational, and chromatin modification, and are also involved in transcriptional interference, variable cleavage, protein modification, and DNA methylation regulation [8, 9]. In addition, lncRNAs can interact with microRNAs (miRNAs) to perform their function. For instance, lncRNA sequences possess miRNA-binding sites, which serve as miRNA endogenous target mimics [10]. In plants, lncRNAs were found to play roles in developmental regulation, reproductive development, photomorphogenesis, Pi homeostasis, fertility, flowering time, immunity, and abiotic or biotic stress responses [5, 11, 12]. In addition, some plant lncRNAs have been studied intensively; for instance, a DROUGHT INDUCED lncRNA (*DRIR*) can facilitate drought and salt tolerance. *DRIR* regulates many genes, including those involved in water transport, abscisic acid (ABA) signaling, and other stress-relief processes [13]. A cold-responsive intergenic lncRNA 1 (CRIR1) was identified from cassava, which is induced by cold treatment. The expression of CRIR1 in cassava regulates a series of cold tolerance genes belonging to a CBF-independent pathway [14]. Cao *et al*. [15] selected ten lncRNAs and silenced them using virus-induced gene silencing to determine their roles in cold stress tolerance in cotton seedlings. One lncRNA, XH123, could confer cold tolerance. The silencing of XH123 results in the decay of chloroplast and elevated cold sensitivity, suggesting that the XH123 was involved in cold tolerance regulation in cotton seedlings. An expression of lncRNAs was compared between two varieties of rice, heat-susceptible SYD2 and heat-tolerant ZS97B, in response to heat treatment. Totally, 231 heat-responsive lncRNA (HRLs) were determined. Among these HRLs, 31 lncRNAs common to SYD2 and ZS97B; however, there were 97 and 103, respectively, to be found in SYD2 and ZS97B. Ten HRLs were found to be linked with five heat tolerance-related QTLs [16]. Until now, some studies of plant lncRNAs on salt stress had been performed, and many salt-responsive lncRNAs had been identified [17–21]. Some investigation of lncRNAs had been performed on the model tree. For instance, Ma *et al*. [22] characterized the roles of lncRNAs in response to salinity in two poplars (*Populus var. Pyramialis* and *Populus euphratica*). In total, 10 531 and 10 646 lncRNAs were respectively identified from these two poplars. Ye *et al*. [23] identified 2988 lncRNAs from 27 RNA-seq datasets in leaves, stems, and roots of *Populus trichocarpa*. Among them, 1183 lncRNAs were differentially expressed by salt. In addition, they further found that salt-responsive lncRNA, Ptlinc-NAC72, induces *PtNAC72* expression by recognizing the tandem element (‘GAAAAA’) in the 5’-UTR of *PtNAC72*. Meanwhile, overexpression of *Ptlinc-NAC72* produced an allergic phenotype under salt stress. However, only a few of the salt tolerance or sensitivity lncRNAs had been characterized, and many of the thousands of lncRNAs identified remain functionally uncharacterized [14]. In addition, there is still a lack of an efficient method for the identification of abiotic stress tolerance lncRNA.

In the present study, the genes and lncRNAs responding to salt stress were identified from the root and leaf tissues of birch (*Betula platyphylla* Suk.). Salt-responsive genes and lncRNAs were identified from birch roots and leaves. Analysis of these genes and lncRNAs suggested that roots and leaves employ different strategies for responding to salinity stress. The biological processes involved in salinity stress had been identified, and the genes associated with salt stress in these processes were determined. We build a method to quickly identify the salt tolerance of lncRNAs. Using this method, some salt-responsive lncRNAs were functionally characterized, and the lncRNAs associated with salt tolerance were determined. A lncRNA conferring high salt tolerance was further characterized. These results may provide helpful information for understanding the function of lncRNAs in salt tolerance in depth.

## Results

### Characterization of the physiological status of birch treated with NaCl

**Figure 1:**
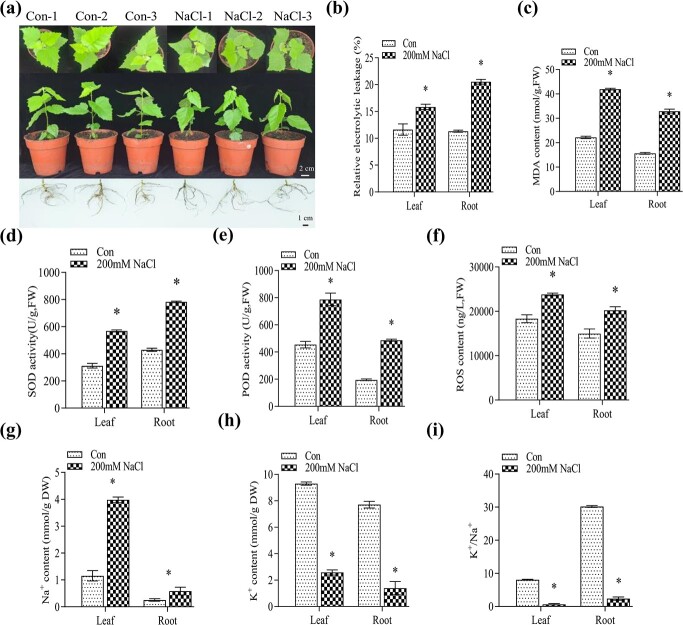
Physiological status of the birch seedling plants used for RNA-seq. **a** The birch growth phenotype under 200 mM NaCl stress conditions or normal conditions. **b** Analysis of electrolyte leakage rate. **c** Analysis of MDA content. **d** Analysis of SOD activity. **e** Analysis of POD activity. **f** ROS content analysis. **g** Na^+^ content analysis. **h** K^+^ content analysis. **i** K^+^/Na^+^ ratio. 200 mM NaCl: Well-watered birch seedlings were irrigated on their roots with 200 mM NaCl solution, and the roots and leaves were separately harvested at 24 h after treatment for analysis. Con: Birch seedlings well-watered with fresh water. Data indicates the mean value of the three biological replicates. The error bar represents the square deviation (SD). Asterisk (^*^) represents a significant (P < 0.05) difference relative to the control plants (Con).

We first determined whether salt treatment induced phenotypic and physiological changes in the birch seedlings. After 24 h of treatment with 0.2 M NaCl, the leaves of birch slightly wilted and drooped (Fig. 1a). The physiological traits, including electrolyte leakage rate, malondialdehyde (MDA) content, activities of superoxide dismutase (SOD) and peroxidase (POD), and reactive oxygen species (ROS) levels were determined after 200 mM NaCl treatment for 24 h. The salt-shock-treated seedlings displayed significantly elevated electrolyte leakage, MDA and ROS contents, and SOD and POD activities in their roots and leaves when compared with the non-stressed birch seedlings. Meanwhile, K^+^ was reduced but Na^+^ was increased in both roots and leaves, resulting in a decreased K^+^/Na^+^ ratio (Fig. 1). These results indicated that salt triggered a series of significant physiological changes (Fig. 1). Therefore, RNA-seq analysis was further performed to investigate the expression of genes and lncRNAs using these plants.

### cDNA library construction and RNA-seq analysis

Four kinds of libraries were constructed, including the birch leaves under normal conditions (Leaf-Con) and salt for 24 h (Leaf-Salt), and the birch roots under normal conditions (Root-Con) and salt for 24 h (Root-Salt). Three biological replicates were conducted; therefore, 12 libraries were constructed. In total, 168 Gb of clean data were generated, and all samples had a Q30 (indicating 99.9% base call accuracy) score greater than 92.35%. The detailed information showed that the quality of high-throughput sequencing could meet the requirements for lncRNA analysis (Table S1, see online supplementary material).

### Determination of the differentially expressed genes from birch responding to salt treatment

**Figure 2:**
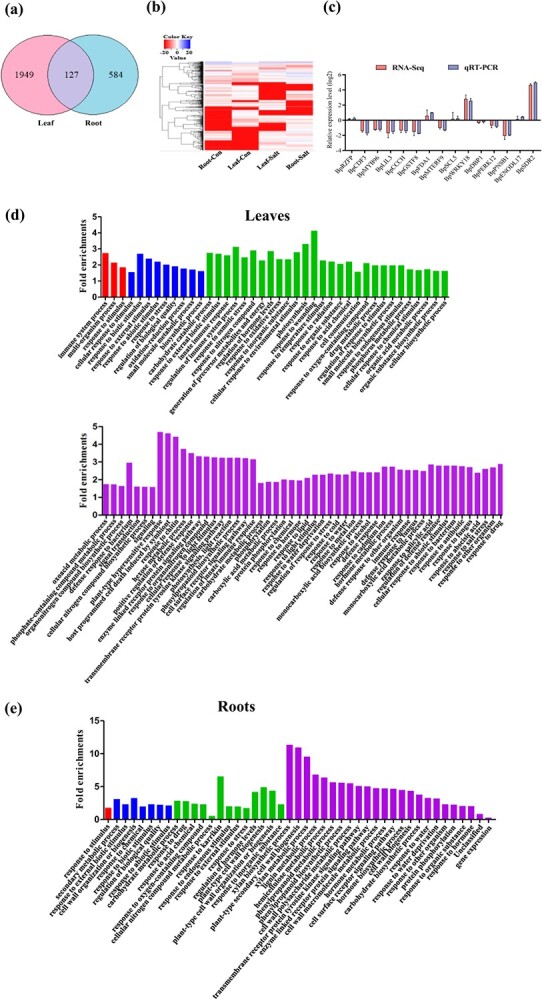
The gene expression of birch in response to salt stress. **a** The distribution of differentially expressed genes (DEG) between the roots and leaves of birch. **b** The expression level of genes in birch leaves and roots responding to salt treatment. Colors indicate the expression levels. **c** Verification of the reliability of RNA-seq data using qRT-PCR. Their Genbank numbers were shown in Table S5 (see online supplementary material). **d** The biological processes that were enriched by salt-responding genes in leaves. **e** The biological processes enriched by DEGs in roots. The red column indicates the third-level GO terms; the blue column indicates the fourth-level GO term; the green column indicates the fifth-level GO term; the purple column indicates the sixth-level GO term.

RNA-seq results showed that there were 2076 and 711 differentially expressed genes (DEGs) respectively identified from the roots and leaves of birch. The distribution of DEGs between roots and leaves was screened. There were only 127 DEG determined in both leaves and roots (Fig. 2a), indicating that gene expression was quite different between the roots and leaves responding to salinity treatment. The expression of the DEGs in leaves and roots responding to salt treatment was displayed as a heatmap (Fig. 2b). To determine whether RNA-seq results were reliable, 16 DEGs were randomly selected and determined their expression using qRT-PCR. The results showed good correlations between RNA-seq and qRT-PCR (Fig. 2c), indicating that RNA-seq result is reliable. Gene ontology (GO) annotation showed that salt stresses highly induced genes involved in ‘response to stimulus (GO:0050896)’ in both leaves and roots (Fig. 2d and e), including its child terms such as ‘response to stress (GO:0006950)’, ‘response to hormone (GO:0009725)’, ‘response to chemical (GO:0042221)’, and ‘response to stimulus (GO:0050896)’. However, in leaves, the genes enriched in ‘response to abiotic stimuli’ was higher than that in the roots. In addition, the terms ‘defense response (GO:0006952)’ and ‘cellular response to stimulus (GO:0051716)’ were highly enriched in leaves, but not in roots when exposed to salt stress (Fig. 2d and e).

There were many differences in the gene expression between roots and leaves. One important phenomenon was that genes involved in cell wall biogenesis or wood development were only enriched highly in roots. For instance, the genes belonging to ‘xylan metabolic process (GO:0045491)’, ‘plant-type secondary cell wall biogenesis (GO:0009834)’, ‘plant-type cell wall organization or biogenesis (GO:0071669)’, ‘plant-type cell wall biogenesis (GO:0009832)’, ‘phenylpropanoid metabolic process (GO:0009698)’, ‘phenylpropanoid biosynthetic process (GO:0009699)’, ‘hemicellulose metabolic process (GO:0010410)’, ‘cell wall polysaccharide metabolic process (GO:0010383)’, and ‘lignin metabolic process (GO:0009808)’ were highly enriched only in roots (Fig. 2d and e). By contrast, genes involved in ‘photosynthesis (GO:0015979)’, ‘phosphorus metabolic process (GO:0006793)’, and ‘cell communication (GO:0007154)’ were only enriched in the leaves (Fig. 2d and e).

### Identification of salt-responsive lncRNAs from birch

**Figure 3:**
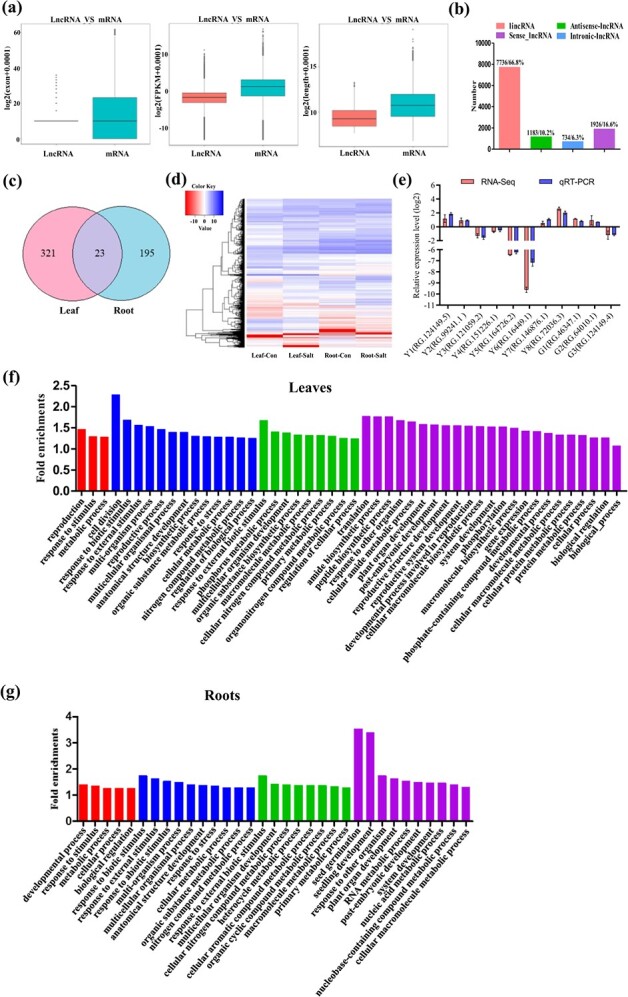
The characteristics of lncRNAs responding to salinity treatment. **a** Comparison of lncRNAs and mRNAs by exon number, expression, and length. **b** Analysis of the types of lncRNAs identified. **c** Overlap of the genes responding to salt between the leaf and root libraries. **d** The transcripts of lncRNAs in different samples. **e** Confirmation of the reliability of RNA-seq data by qRT-PCR; the Genbank numbers of the lncRNAs were included in Table S3 (see online supplementary material). **f** GO analysis of the genes targeted by salt-responsive lncRNAs in leaves. **g** GO annotation of the genes targeted by salt responding lncRNAs in roots. The red column indicates the third-level GO term; the blue column indicates the fourth-level GO term; the green column indicates the fifth-level GO term; the purple column indicates the sixth-level GO term.

In total, 11 579 lncRNAs were identified. The identified lncRNAs and mRNAs from both leaves and roots were compared together. The results showed that most mRNAs have more exons than lncRNAs, while mRNAs are generally longer than lncRNAs, and their transcription levels were higher than those of lncRNAs (Fig. 3a). The numbers of different types of lncRNAs were analysed, and lincRNAs holding the maximum proportion, then sense lncRNAs and antisense lncRNAs; intronic lncRNAs accounting for the lowest percentage (Fig. 3b). The salt-responsive lncRNAs were determined by comparing Leaf-Salt *vs*. Leaf-con and Root-Salt *vs*. Root-Con. There were 190 and 154 lncRNAs that were, respectively, up- and down-regulated by salt stress in leaves. There were 153 and 65 salt-responsive lncRNAs that were respectively up- and down-regulated by salt stress in birch roots (Table S4, see online supplementary material). There were only 23 salt-responsive lncRNAs present in both leaves and roots (Fig. 3c), indicating that roots and leaves have quite different lncRNA expression patterns responding to salinity treatment. Moreover, the salt-responsive lncRNAs in leaves and roots are quite different responding to salt stress (Fig. 3d). To confirm the expression of these lncRNAs responding to salt stress, 11 lncRNAs were selected randomly and qRT-PCR was performed to analyse their expression responding to salt stress. There were good correlations between the qRT-PCR and RNA-seq results (Fig. 3e), indicating that RNA-seq is reliable.

### Analysis of the target genes of lncRNAs

The target genes regulated by salt-responsive lncRNAs were identified, and these salt-responsive lncRNAs were predicted to regulate 5259 target genes. Among the predicted target genes, *trans*-regulation genes only accounted for 17% (894 genes), and most of them were predicted as *cis*-regulatory target genes; however, 62 target genes were found to be both *cis*-regulated and *trans*-regulated (Table S4, see online supplementary material).

The biological processes enriched by the genes targeted by salt-responding lncRNAs were annotated using GO analysis (Fig. 3f and g). In both leaves and roots, ‘metabolic process (GO:0008152)’, ‘developmental process (GO:0032502)’, and ‘cellular process (GO:0009987)’ were enriched significantly. Additionally, the child’s terms of metabolic process, including ‘nitrogen compound metabolic process (GO:0006807)’, ‘response to stimulus (GO:0050896)’, ‘organic substance metabolic process (GO:0071704)’, and ‘primary metabolic process (GO:0044238)’ were all highly enriched both in roots and leaves (Fig. 3f and g).

We further compared the enriched processes of the lncRNAs target genes between the leaves and roots. The enriched processes were similar in leaves and roots. The main differences were that ‘reproduction (GO:0000003)’ and ‘phosphorylation (GO:0016310)’ were highly enriched in leaves, but not in roots. However, the ‘nucleobase-containing compound metabolic process (GO:0006139)’ was only enriched in roots (Fig. 3f and g).

### Building a quick method for characterization of lncRNAs involved in salt tolerance

**Figure 4:**
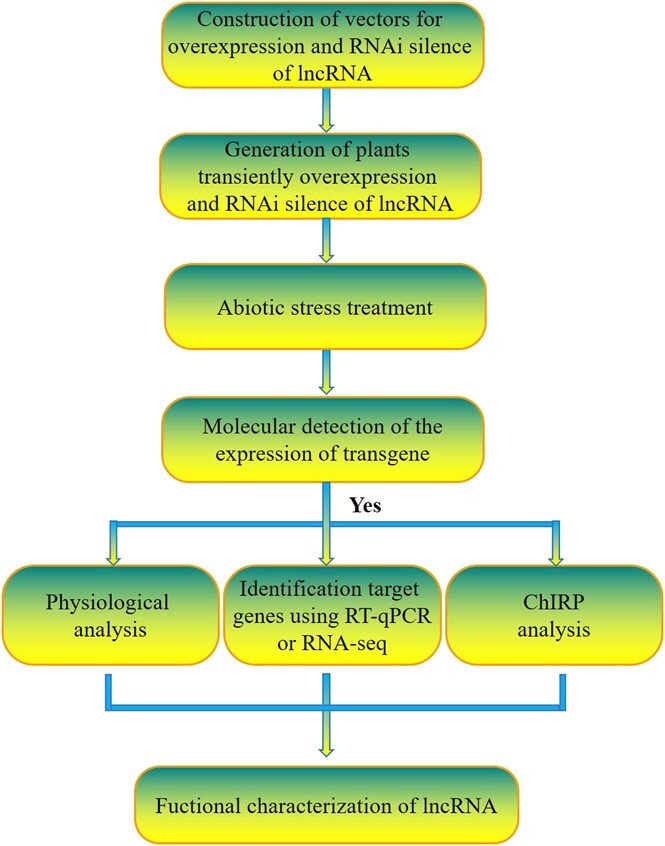
The procedure of quick determination of abiotic stress tolerance of lncRNA. The target lncRNA was respectively cloned into overexpression and RNA interference (RNAi) vectors, and transient transformation was conducted to generate plants overexpressing or knocking-down the target lncRNA. The transformed plants were then treated with abiotic stress, such as salt stress; after treatment, the expression of target lncRNA was determined to study whether the transient transformation was successfully performed. Then, physiological traits involved in abiotic stress tolerance were compared to determine if lncRNA could confer abiotic stress tolerance, sensitivity or not. Also, the target genes of lncRNA or ChIRP can be analysed by using these transiently transformed plants.

To investigate whether these salt-responsive lncRNAs are actually involved in salt tolerance, a method to identify salt tolerance based on transient genetic transformation [24, 25] was built, and this method enables the analysis of lncRNA through gain- and loss-of-function ways. The procedure of this method was as follows (Fig. 4):

(i) construction of the vectors for overexpressing and RNA interfering with the aim lncRNA; at the same time, an empty vector (either overexpression or RNA interfere vector is acceptable) was used for control;
(ii) transient genetic transformation was performed for generating the birch plants with transient overexpressing and knocking-down lncRNA, and the birch plants transiently transformed with the pROK2 (empty vector) were generated serving as the control plants;
(iii) abiotic stress treatment was carried out on the transiently transformed plants based on the experiment requirement;
(iv) molecular detection was performed to determine whether the transient overexpression or knock-down plants had been successfully generated;
(v) physiological traits involved in abiotic stress tolerance were analysed, and abiotic stress tolerance was determined by comparing these physiological traits between the plants transient overexpressing and knocking-down of lncRNA, and the control plants; and
  (a) if identification of target genes of lncRNA is needed, RNA-seq or qRT-PCR can be performed on these transiently overexpressing, knocking-down target lncRNA and the control plants;
  (b) ChIRP analysis can be performed using the transiently overexpressing target lncRNA plants.

**Figure 5:**
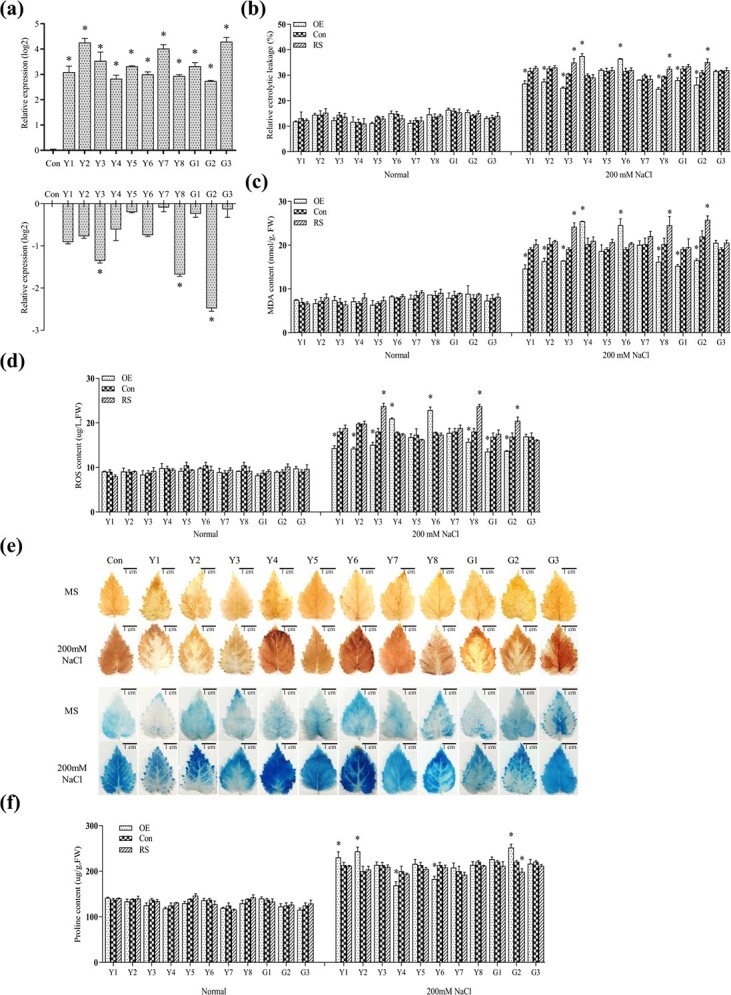
Analysis of the salt tolerance conferred by lncRNAs. **a** Determination of the transgene expression. **b** Analysis of electrolyte leakage rates. **c** Analysis of MDA content. **d** Analysis of ROS content. **e** Detection of cell death and ROS accumulation in birch leaves using Evans blue and DAB staining. **f** Proline content analysis. Three biological replications were carried out. The data indicates the mean of different replications. Con: birch plants were transiently transformed with an empty pROK2 vector; OE: birch plants transiently overexpressing the lncRNA; RS: birch plants transiently interfering (knocking-down) lncRNA. Compared with Con plants, some physiological traits in OE plants changed significantly but were not changed in RS plants. This was because the expression of the studied lncRNAs could not be interfered with by RNAi in the transgenic plants. The Genbank accession numbers of these lncRNAs are included in Table S3 (see online supplementary material). Error bars indicate the SD. An asterisk (^*^) represents a significant (*P* < 0.05) difference relative to control plants (Con).

The 11 randomly selected salt-responsive lncRNAs were cloned separately into pROK2 for overexpression (OE) or cloned into pFGC5941 for RNAi-mediated expression silencing (RS). The expression of the lncRNA was first determined in the transient transformation plants. The transcripts of the studied lncRNAs in the OE plants were highly elevated (Fig. 5a), indicating that the transgenic plants transiently overexpressing lncRNAs had been successfully generated, and could be used for further study. However, only three lncRNAs were significantly downregulated in transgenic plants transformed with pFGC5941 for RNAi-mediated knockdown (Fig. 5a). This could be due to the reason that RNAi can interfere with the expression of lncRNAs located only in the cytoplasm; however, many lncRNAs are also or only located in the nucleus, resulting in failure of RNAi. Therefore, only the three transgenic plants with knockdown of lncRNAs could be used to analyse lncRNA function.

**Figure 6:**
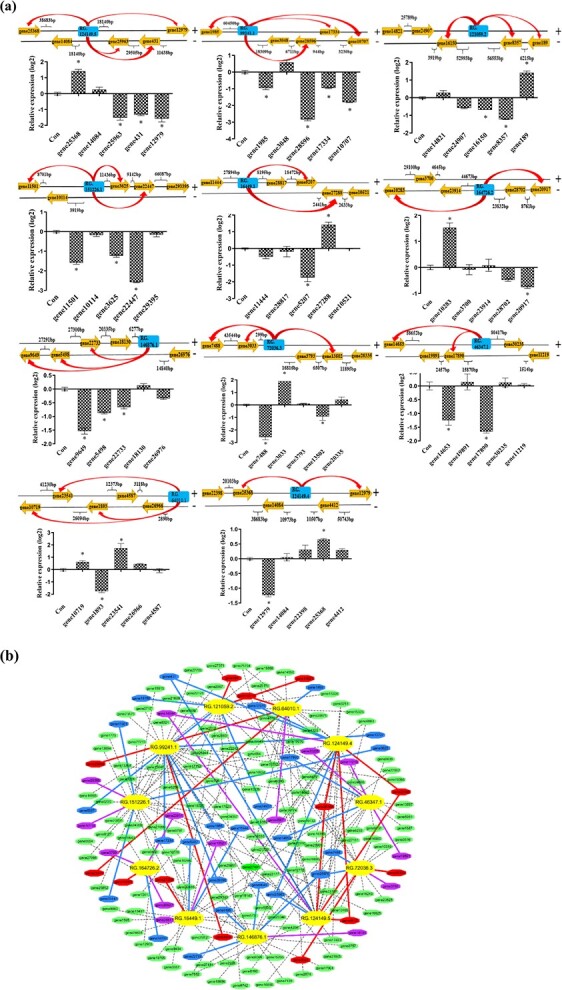
Confirmation of the genes regulated by lncRNAs. **a** Confirmation of the lncRNA target genes using qRT-PCR. The expression of genes between birch transiently overexpressing lncRNAs and their controls (transiently transformed with vector pROK2) were compared using qRT-PCR. The positions of the lncRNAs and their target genes are shown in the upper part of the chart. The blue frame indicates the position of the lncRNA, and the bright yellow arrow indicates the position of their predicted target genes. Red lines point to the genes actually regulated by the lncRNA according to qRT-PCR. Asterisk (^*^) represents a significant difference with fold change >1.2 (*P* < 0.05, *t*-test). **b** Prediction and verification of the target genes regulated by 11 studied lncRNAs. The relationships between the predicted genes and the 11 lncRNAs are shown. Bright yellow inside the circle indicates the lncRNAs. The green dots outside the circle indicate the target genes regulated by the lncRNAs. Red dots and lines indicate the target genes induced by lncRNAs that were confirmed using qRT-PCR. Blue dots and lines indicate the target genes inhibited by lncRNAs, which were confirmed using qRT-PCR. The purple dots and lines indicate the genes that were not regulated by the studied lncRNA based on the qRT-PCR results. Their Genbank numbers are shown in Table S5 (see online supplementary material).

To determine the salt tolerance of the plants overexpressing or knocking-down for these lncRNAs, the physiological changes related to abiotic stress tolerance were measured, including MDA content, ROS content, and the electrolytic leakage rate (Fig. 5b–d). Among the studied lncRNAs, the expression levels of *LncY5*, *LncY7*, and *LncG3* were unrelated to the MDA content, ROS level, and electrolytic leakage rate (Fig. 5b–d). In addition, DAB and Evans blue staining indicated that overexpression of these lncRNAs failed to alter H_2_O_2_ accumulation and integrity of cell membrane (Fig. 5e). Therefore, these three lncRNAs did not play roles in salt tolerance.

However, overexpression of lncRNAs *LncY1*, *LncY2*, *LncY3*, *LncY8*, *LncG1*, and *LncG2* decreased ROS contents, MDA contents, and electrolytic leakage rates (Fig. 5b–d). Consistently, the knockdown of *LncY3*, *LncY8*, and *LncG2* increased the MDA and ROS contents and the electrolytic leakage rates (Fig. 5b–d). In addition, overexpression of these lncRNAs reduced H_2_O_2_ accumulation and cell membrane integrity according to DAB and Evans blue staining (Fig. 5e). Therefore, overexpression of these lncRNAs increased salt tolerance, and *LncY3*, *LncY8*, and *LncG2* are salt tolerance-related lncRNAs.

Conversely, overexpression of lncRNAs *LncY4* and *LncY6* increased the MDA and ROS contents and enhanced the electrolytic leakage rates (Fig. 5b–d). Meanwhile, Evans blue and DAB staining indicated that overexpression of these lncRNAs increased H_2_O_2_ accumulation and induced cell membrane damage (Fig. 5e). These results suggested that *LncY4* and *LncY6* are sensitive to salt stress.

In addition, the RS plants whose target lncRNA expression did not respond to RNAi all showed similar MDA levels, ROS contents, and electrolytic leakage rates to the control plants (Fig. 5b–e). These results further confirmed that the physiological changes were due to the changed expression level of lncRNAs.

To further study the function of these lncRNAs, the proline contents of the transiently transformed plants were measured (Fig. 5f). Alteration of the expression levels of lncRNAs that failed in conferring salt tolerance also did not affect the proline content. Among the lncRNAs conferring salinity tolerance, the expression of *LncY3* and *LncY8* did not alter proline contents. Overexpression of *LncY1*, *LncY2*, and *LncG2* all significantly increased the proline content. By contrast, *LncY4* and *LncY6* are salt-sensitive lncRNAs, and their overexpression significantly decreased the proline content (Fig. 5f). These results suggested that *LncY1*, *LncY2*, *LncY4*, *LncY6*, and *LncG2* could regulate salt tolerance by controlling proline biosynthesis; however, *LncY3* and *LncY8* probably improve salt tolerance via a proline-independent process.

### Determination of the genes regulated by lncRNA

To study whether the predicted target genes were really regulated by lncRNAs located in close proximity, we studied the predicted target genes of 11 studied lncRNAs by comparing the expression of the plants transiently overexpressing the lncRNA and the control plants (transient transformation of empty pROK2) using qRT-PCR (Fig. 5a). The qRT-PCR results indicated that only part of the potential target genes was significantly regulated (P > 0.05, fold change >1.2) by the lncRNAs, suggesting that they could be the target genes of the lncRNAs (Fig. 6a). In addition, many target genes of lncRNAs were transcription factors (TFs), implying that these TFs are involved in salt response mediated by these lncRNAs. Fig. 6b shows the relationships among the 11 lncRNAs and their predicted target genes based on qRT-PCR results.

### Identification of the genes regulated by *LncY1*

Among the 11 studied lncRNAs, *LncY1* displayed highly decreased MDA and ROS contents, and reduced electrolyte leakage when compared with the other lncRNAs (Fig. 5), suggesting that it improves salt stress tolerance to a greater degree than the other lncRNAs; therefore, *LncY1* was selected for further study.

As the target genes of *LncY1, BpMYB96* (Gene25368 Myb-like DNA-binding domain) and *BpCDF3* (Gene12979 DOF domain) had been predicted by bioinformatics prediction. In addition, qRT-PCR indicated that *BpMYB96* and *BpCDF3* could be induced and inhibited, respectively (Fig. 6a). The function of these two TFs was further determined.

**Figure 7:**
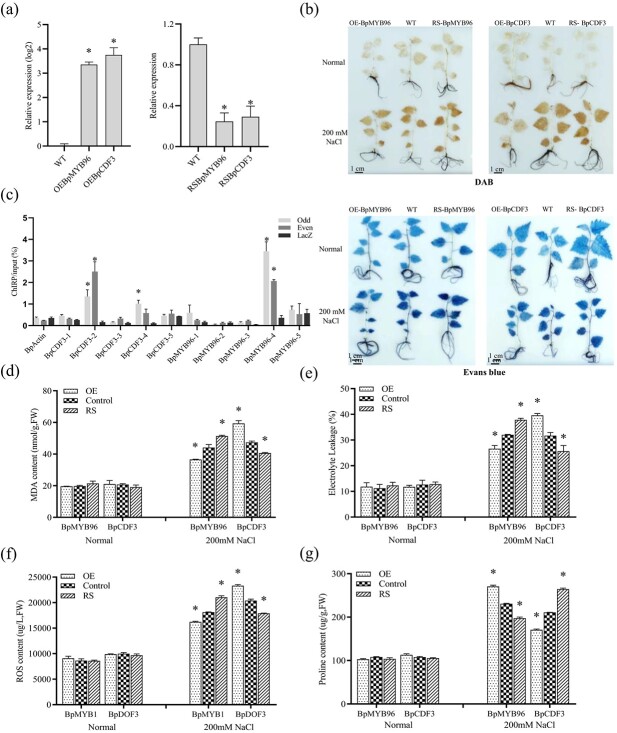
*LncY1* regulates *BpMYB96* and *BpCDF3* to confer salt tolerance. **a** Detection of the transgenes in the plants that transient overexpress or knock-down the TFs using qRT-PCR. **b** Comparison of the phenotype among the studied plants by using Evans blue and DAB staining. The OE, RS, and WT plants were respectively stained with DAB and Evans blue. **c** Chromatin isolation by RNA purification (ChIRP) analysis of the binding of *LncY1* to promoters of *BpMYB96* and *BpCDF3*. Input, input sample. Odd, samples hybridized with odd probes; Even, samples hybridized with even probes; LacZ, LacZ-DNA probes were used as the negative control. **d** Analysis of MDA content. **e** Analysis of electrolyte leakage. **f** Analysis of ROS content. **g** Assay of proline content. Normal: Under normal growth conditions. Control: Birch plants transiently transformed with an empty pROK2 serving as the control. NaCl: 0.2 M NaCl treatment. OE: Birch plants transient overexpressing TFs (BpMYB96 or BpCDF3); RS: Birch plants transiently RNAi-silenced for lncRNA. Three independent biological replications were performed. The Genbank numbers of these genes are included in Table S3 (see online supplementary material). The error bar represents the square deviation (SD). Asterisks (^*^) represents significant (*P* < 0.05) difference relative to the control birch plants (Control).

*BpMYB96* and *BpCDF3* were transiently RNAi-silenced or overexpressed in birch for gain- and loss-of-function analysis. qRT-PCR was first performed to study the expression of TFs among the control, the transiently RNAi-silenced and overexpressed plants. All these TFs were significantly induced or knocked down, suggesting that they can be used for gain- and loss-of-function studies (Fig. 7a). To determine whether *BpMYB96* and *BpCDF3* can confer salt tolerance, the plants with transient overexpression and RNAi silence of *BpMYB96* and *BpCDF3* were treated with salt stress. DAB and Evans blue staining were performed to study whether NaCl treatment had changed physiological changes in these plants. The results showed all the plants displayed similar staining phenotype under normal growth conditions (Fig. 7b). When exposed to salt treatment conditions, the plants with knock down of *BpMYB96* or *BpCDF3* both showed the highest H_2_O_2_ accumulation and cell membrane damage, followed by WT plants, and the plants overexpressing *BpMYB96* or *BpCDF3* both displayed the lowest H_2_O_2_ accumulation and cell membrane damage. These results suggested that NaCl treatment had substantially changed the physiological traits involved in stress (Fig. 7b).

To study whether *BpMYB96* and *BpCDF3* were directly regulated by *LncY1*, ChIRP was performed. The promoter sequence (2000 bp upstream of ATG translation initiation codon) was divided into five equal sites for ChIRP-qPCR amplification. The results indicated that the truncated promoters of the studied TFs were enriched significantly by ChIRP either using ‘Even’ or ‘Odd’ probes, suggesting that these two TFs were regulated by *LncY1* through the way that their promoters interacted with *LncY1* (Fig. 7c).

Compared with the control plants, overexpression of *BpMYB96* resulted in decreased MDA and ROS contents, and decreased electrolyte leakage rates; meanwhile, the plant with knockdown of *BpMYB96* displayed increased electrolyte leakage and elevated MDA and ROS contents (Fig. 7c–e). These results indicated that *BpMYB96* confers tolerance to salt stress. Conversely, overexpression of *BpCDF3* resulted in elevated ROS and MDA contents, and increased electrolyte leakage rates, while their knockdown had the opposite effect (Fig. 7c–e), which suggested that expression of *BpCDF3* leads to decreased salt tolerance. The expression of *LncY1* increased the proline level; therefore, we also determined whether these TFs regulated by *LncY1* are involved in proline biosynthesis. Overexpression of *BpMYB96* or knockdown of *BpCDF3* both resulted in increased proline contents (Fig. 7f). Conversely, down-regulation of *BpMYB96* or increased expression of *BpCDF3* both resulted in decreased proline contents (Fig. 7f). Importantly, the salt stress tolerant TF *BpMYB96* was induced by *LncY1*; meanwhile, the salt stress-sensitive TF *BpCDF3* was inhibited by *LncY1*. These results indicated that *LncY1* could improve salt tolerance by up-regulating *BpMYB96* and down-regulating *BpCDF3*.

## Discussion

### Birch roots and leaves employed different ways for adaption of salt stress

The salt-responsive genes and lncRNAs were respectively identified in the leaves and roots of birch. Through the analysis of the genes and lncRNAs, we found that the number of DEGs in the leave tissue was more than 3-fold of the DEGs in the root tissue. In addition, only 127 DEGs were found in both leave and root tissues (Fig. 2a). Additionally, the lncRNAs responding to salt were also different in roots and leaves (Fig. 3c), indicating that different ways for adaption of salt stress were employed in leaves and roots.

Previous studies showed that salt stress induces increased deposition of lignin and suberin in endodermal and exodermal cells, which improves salt tolerance [26]. Many genes involved in the cell wall or wood development also play roles in salt tolerance [27–29]. The DEGs were preferentially enriched in processes related to cell wall biogenesis and wood development in roots rather than in leaves (Table S2, see online supplementary material), suggesting that birch roots might improve salt tolerance by enhancing the processes related to cell wall biogenesis and wood development, which would increase the deposition of lignin and change the cell wall composition of specific root cell types, leading to increased salt stress tolerance.

Compared with roots, the DEGs in the leaves were preferentially enriched for the ‘small molecule metabolic process’ (GO:0044281) (Fig. 2d and e), indicating that this process was enhanced in leaves in response to salt treatment. Small molecule metabolic substances, including proline, soluble sugars and glycine betaine all are important for salt tolerance in plants, indicating that leaves might adapt to salt stress by increasing the osmotic potential. In addition, photosynthesis (GO:0015979) and phosphorylation (GO:0016310) were also preferentially enriched in leaves, both of which are involved in salt tolerance [30–32]. These results indicated that salt stress induces small molecule metabolic process, photosynthesis and phosphorylation in leaves, and these enhanced processes contribute to salt tolerance in birch.

### Building a quick method for identification of abiotic stress tolerance of lncRNA

Previously, we had built a method for quick determination of abiotic stress tolerance and analysis of regulatory relationships [24, 33, 34]. This method is based on the transient transformation technology we constructed and could obtain the plants with transient overexpressing or knocking-down of plants. During the period of gene transient expressing or knocking-down, these transiently transformed plants can be used for physiological and molecular analysis as stably transformed plants. In the present study, this method is also used in characterizing the function of lncRNAs, which could be used in abiotic stress tolerance analysis, identification of the potential target genes of lncRNAs and even could be used for ChIRP and may have a wide application in plant lncRNA investigation.

### LncRNAs mediated salt tolerance in birch

LncRNAs are important regulators in different biological processes. Plants accumulate nitrogen-containing compounds (NCCs), including amides, amino acids, and polyamines under salt stress conditions. The NCCs play key roles in improving tolerance to abiotic stress by scavenging free radicals, storage of nitrogen, osmotic adjustment, maintaining cellular pH, detoxification of cells, and protecting cellular macromolecules [35]. The target genes of salt-responsive lncRNA were enriched significantly in the ‘nitrogen compound metabolic process (GO: 0006807)’ in both leaves and roots (Fig. 3), indicating that NCCs should be highly accumulated in roots and leaves of birch to improve salt tolerance. In addition, the predicted target genes regulated by salt-responsive lncRNAs from roots and leaves were both enriched significantly in ‘response to stimulus (GO: 0050896)’ and these genes were associated with improving salt stress tolerance (Fig. 3), suggesting that lncRNAs regulate these genes to improve salt tolerance. Therefore, ‘response to stimulus (GO: 0050896)’ and ‘nitrogen compound metabolic process (GO: 0006807)’ are regulated by lncRNAs to facilitate salt tolerance in birch.

### Investigation of the genes regulated by lncRNAs

LncRNAs usually regulate their target genes through *cis*- and *trans*-regulation [36]. In the present study, we performed qRT-PCR to confirm the potential target genes of the salt-responsive lncRNAs. According to the qRT-PCR results, only part of the potential target genes was highly differentially regulated (fold change >2.0) by lncRNAs. This may be explained as follows. First, they were not actually the target genes of these lncRNAs. Second, lncRNAs can regulate their target genes only in certain specific tissues or under certain conditions. Third, these predicted target genes could be regulated by lncRNAs, but not to a high degree. Indeed, some target genes were significantly induced or inhibited (P > 0.05) by these lncRNAs, as predicted, but were not highly differentially regulated (Fig. 6a). Therefore, we used the fold change >1.2 as the criterion for highly differentially regulated to determine whether there was a regulatory relationship between the lncRNAs and the genes. However, whether this criterion was appropriate requires further study.

### The transient transformation method could be used in characterizing the function of lncRNA

In the present study, we used the transient transformation method to study the function of lncRNA. This method had been used in characterizing the function of genes responding to abiotic stress [33, 37]. Here, we characterized the function of lncRNA in salt tolerance using this method. This method can not only analyse the function of lncRNA involved in salt tolerance but also can identify their target genes quickly. Therefore, it is a powerful technology for characterizing lncRNAs involved in abiotic stress. Of course, some of the lncRNAs obtained may not be full-length transcripts; however, their overexpression resulted in an altered salt stress response. Therefore, large-scale identification of lncRNAs related to salt tolerance using this transient transformation method is practicable. False negatives may be identified among salt tolerance-related lncRNAs because some truncated lncRNAs might be too short to exert their function. However, this could be resolved using advanced high-throughput sequencing.

### *LncY1* confers salt tolerance by regulating the TFs *BpMYB96* and *BpCDF3*

The target gene prediction indicated that *LncY1* could directly regulate two TFs, *BpMYB96* and *BpCDF3*, whose encoding genes are located adjacent to *LncY1* in the genome. At the same time, both *BpMYB96* and *BpCDF3* can be induced or inhibited by *LncY1,* respectively (Fig. 6a), suggesting that these two genes are the target genes of *LncY1*. ChIRP study indicated that *LncY1* binds to the promoters of *BpMYB96* and *BpCDF3* (Fig. 7b). Taken together, *LncY1* induces the expression of *BpMYB96* and inhibits the expression of *BpCDF3* by binding to their promoters (Fig. 7a). In addition, overexpression of *BpMYB96* significantly reduced the contents of MDA and ROS and electrolyte leakage rate, but increased proline content (Fig. 7c–f). On the contrary, knocking down of *BpCDF3* significantly reduced the contents of MDA and ROS and decreased electrolyte leakage rate, but improved proline content (Fig. 7c–f). Correspondingly, overexpression of *LncY1* also significantly decreased MDA and ROS content, and reduced electrolyte leakage rate, but improved proline content (Fig. 5). Taken together, these results suggested that *LncY1* respectively induces and inhibits the expression of *BpMYB96* and *BpCDF3*, resulting reduced membrane lipid peroxidation and ROS accumulation, reduced cell membrane damage, and improved osmotic potential (Fig. 5 and 7), which finally improve salt tolerance.

## Conclusions

**Figure 8:**
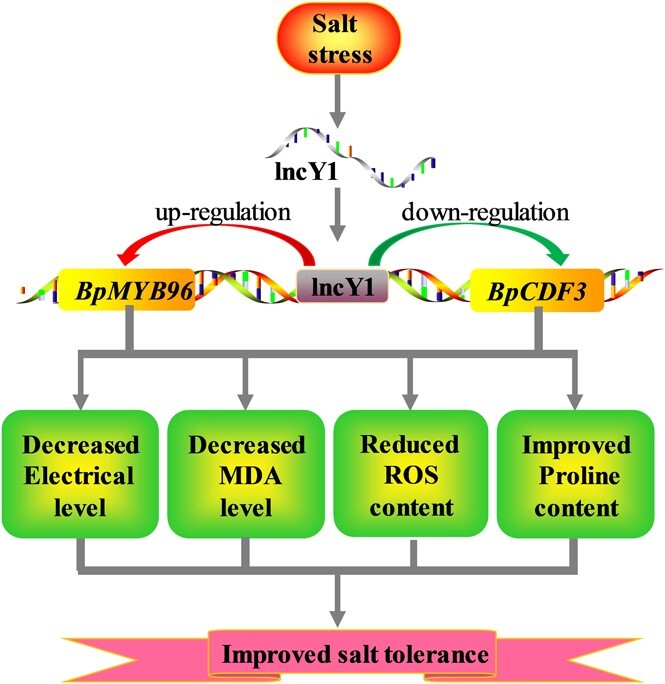
Model of the regulatory network of *LncY1* responding to salt treatment. Salt treatment significantly up-regulates the expression of *LncY1*. Activated *LncY1* regulates the salt stress by inhibiting and up-regulating the expression of *BpCDF3* and *BpMYB96*, respectively, resulting in reduced ROS accumulation, Electrolyte levels, MDA content, and increased proline level. These physiological changes finally lead to improved salt tolerance.

We found that in birch roots, the processes involved in wood development and cell wall biogenesis were highly activated and should play important roles in salinity stress response. However, the genes involved in ‘response to stimulus’ play their roles in salinity tolerance mainly in leaf tissue rather than in root tissue. Furthermore, lncRNAs were found to mediate salt stress tolerance mainly by enhancing the ‘nitrogen compound metabolic process’ and ‘response to stimulus’. A method for quick identifying abiotic stress tolerance of lncRNAs was built. Using this method, five salt tolerance and three salt sensitive lncRNAs were identified. In addition, a working model was proposed for salt tolerance mediated by *LncY1*. Salinity induces the expression of *LncY1*, and then the induced *LncY1* up-regulates the expression of *BpMYB96* and down-regulates the expression of *BpCDF3*. Up- and down-regulation of these two TFs triggered significant physiological changes in birch plants, including reduced electrolyte leakage rate, decreased ROS accumulation, reduced MDA content, and elevated proline content, which ultimately improved salt stress tolerance in the birch plant (Fig. 8).

## Materials and methods

### Plant treatment and growth conditions

Birch plants were seeded in soil and grown in a greenhouse under conditions of 400 μmol/m^2^/s light intensity, 16 h/8 h light and dark cycle, and relative humidity of 65–75%. The birch plants (10-week-old) were watered with water supplied with 0.2 M NaCl for 24 h, and the plants watered with fresh water were served as the control. Each treatment contained at least 20 seedlings. Three biological replicates were carried out. The leaves and roots of birch were separately harvested for RNA-seq study.

### Illumina sequencing and identification of lncRNAs

Total RNAs from different samples were extracted using a cetyltrimethylammonium bromide (CATB) method. To remove rRNA from total RNA, Epicentre Ribo-Zero™ Kit (Epicentre, Madison, WI, USA) was used. The cDNA library was constructed using NEBNext Ultra Directional RNA Library Prep Kit for Illumina (NEB, Ipswich, MA, USA). Sequencing of the libraries was carried out on the Illumina HiSeq 4000 platform (Illumina, San Diego, CA, USA). Low-quality reads were first removed, including adaptor sequence, reads containing N > 10% (N represents a base that could not be determined), and sequences with Q ≤ 10 to generate the clean reads. The HISAT2 program [38] was employed to map the reads to the birch genome (https://phytozome-next.jgi.doe.gov/info/Bplatyphylla_v1_1) [9]. Transcripts were assembled according to the annotation of the birch genome using StringTie [39]. Each transcript quantified its expression as the FPKM value using StringTie. For annotating these assembled transcripts, the gffcompare program was applied. LncRNAs were identified from the transcripts without annotation. The transcripts more than 200 nt in length were selected as the candidates of lncRNA. The programs of CPC/CNCI/Pfam/CPAT were together applied to select the lncRNA [40–44]. Anti-sense lncRNA, lincRNA, intronic lncRNA and sense lncRNA were screened using the cuffcompare program. The DEGs and differentially expressed lncRNAs were determined with the criterion of fold change >2 (*P*-value <0.01, *t*-test). Three biological repetitions were carried out. The above experiments were performed at Beijing Biomarker Technologies Corporation (Beijing, China). Gene ontology (GO) annotation (http://www.geneontology.org/) was carried out on the DEGs. All sequencing data were submitted to the SRA database (accession number PRJNA790472).

### Prediction of the target genes of lncRNAs

The target genes of lncRNA were predicted using two methods. One prediction was performed based on the positional relationships between lncRNA and gene, and the genes within 100 kb location of a lncRNA were considered as potential target genes with *cis*-acting [45]. For the trans-acting target gene prediction, lncTar software was employed to determine the binding free energy of lncRNA and mRNA pairs with the threshold (<−0.1) predicated as the *trans*-acting [46].

### Construction of expression vectors

The sequence of the lncRNA was inserted into pROK2 controlled by CaMV35S promoter for constructing the lncRNA expression vector. For constructing gene expression vectors, the coding sequence (CDS) of the genes was respectively cloned into pROK2, controlled by the promoter of CaMV35S. For constructing RNA interference (RNAi) vector, a truncated CDS of a gene or the lncRNA sequence was cloned in the reverse and forward orientations into pFGC5941 by forming an inverted repeat sequence for RNAi interference with the gene or lncRNA expression. The primers were included in Table S15 (see online supplementary material).

### Determination of salt stress tolerance of genes or lncRNAs

Salt tolerance of lncRNAs or genes was characterized according to transient genetic transformation according to the description of Zang *et al*. [24]. In brief, whole birch plants were soaked in transformation solution [1/2 MS (pH 5.7) + 0.02% Tween 20 (v/v) +5% (w/v) sucrose +120 μM acetosyringone +10 mM CaCl_2_ + 0.001% (w/v) DTT + 0.6 OD *Agrobacterium tumefaciens*] with 90 rpm rotation at 22°C for 2 hours. The birch plantlets then were washed with fresh deionized water quickly. Plantlets were grown on MS solid culture medium [MS + 2.5% (w/v) sucrose, pH 5.8] recovery for 48 h. Then the transformed plantlets were moved into solid MS medium supplied with 0.2 M NaCl for salt treatment for 24 h. The transiently transformed plantlets grown on a solid MS medium without NaCl were used as the control conditions. Plantlets transiently transformed with empty pROK2 vector were used as the control samples (Con).

Staining with 3,3-diaminobenzidine (DAB) and Evans blue was carried out according to the methods of Yang *et al.* [47]. Electrolyte leakage rate was measured according to the approach described by Dionisio-Sese and Tobita. [48]. Malondialdehyde (MDA) was analysed following the approach described by Madhava Rao and Sresty. [49]. Activities of SOD and POD were analysed as the previous description [50]. The ROS content was measured with a ROS ELISA kit (Senbeijia Bio. Co., Nanjing, China). The method of Bates *et al.* [51] was used to determine proline content.

### Verification of the genes regulated by lncRNAs

The transgenic birch transiently overexpressing lncRNAs were employed to determine the expression of genes potentially regulated by lncRNAs, and the plantlets transiently transformed with empty pROK2 vector were used as the control. The expression of genes between the control plants and the plants overexpressing lncRNA was compared using qRT-PCR.

### Real-time PCR

Total RNA without DNA contamination (about 2 μg) was reverse transcribed into cDNA using oligo(dT) as a primer and was performed with a PrimeScript™ RT reagent Kit (Takara, Shiga, Japan) in a 10 μl volume. The cDNA was diluted to 100 μl with ultrapure water serving as a PCR template. The real-time PCR reaction system included 0.5 μM of each forward or reverse primer, 2 μl of cDNA template, and 10 μl of SYBR Premix Ex Taq™ (Takara). The tubulin (GenBank number: FG067376) and ubiquitin (GenBank number: FG065618) were used as the internal controls. The thermal profiles were: 94°C 30 s; 40 cycles of 94°C for 15 s, 58°C for 30 s, and 72°C for 45 s, and were carried out on a qTower 2.2 (Analytik Jena AG, Jena, Germany). The relative expression was determined using 2^-ΔΔCT^ method with three biological replicates [52]. All the primer sequences and the GenBank numbers studied ware included in Table S5 (see online supplementary material).

### Chromatin isolation by RNA purification (ChIRP)

A set of probes based on the sequence of *LncY1* for ChIRP were designed, and the interval of each probe was approximately 100–150 bp. In total, five probes were designed and named from 1 to 5. The anti-sense DNA probes labelled with Biotin-TEG at the 3′ end were synthesized (Shanghai Sangon Biotechnology Co., Ltd). The five probes were classified into two groups based on their position. One group was ‘odd’ and contains probes 1, 3, and 5. The ‘even’ group contains probes 2 and 4. ChIRP assays were conducted following Chu *et al*. [53]. Three biological replicates were performed. The LacZ cDNA probe is employed as the negative control because it is not a homologue with any RNA sequence from birch. Each DNA sample acquired was analysed by qPCR. All the probes and primers were included in supplementary in Table S5 (see online supplementary material).

### Statistical analysis

The data was assayed using one-way analysis of variance (ANOVA), and statistical assay was conducted with the Statistical Package for the Social Sciences (SPSS 22, IBM Corp., Armonk, NY, USA) and *P* < 0.05 was considered as statistical significance.

## Acknowledgements

This work was supported by the Xingliao Talent Plan Project XLYC1902007, and Funds for Guiding Local Scientific and Technological Development by the Central Government 202JH6/10500071. We thank Northeast Forestry University, State Key Laboratory of Tree Genetics and Breeding for Arid Areas’ shared instrument platform.

## Author contributions

Y.W. conceived the original screening and research plans; H.Z. designed the experiments and analysed the data; Y.J. and Y.N. provided technical assistance to H.Z.; Y.W. and Y.J. conceived the project and wrote the article with the contributions of all the authors.

## Data availability

All the data used to support the findings of this study are available from the corresponding author upon reasonable request.

## Conflict of interest

The authors declare no competing interests.

## Supplementary data

Supplementary data is available at *Horticulture Research* online.

## Supplementary Material

Web_Material_uhac277
